# Supplementary material for: Dynamics of cellular states of fibro-adipogenic progenitors during myogenesis and muscular dystrophy
Source: Nat Commun. 2018 Sep 10;9:3670. doi: 10.1038/s41467-018-06068-6 (PMC6131350; doi:10.1038/s41467-018-06068-6)
Supplement: Supplementary file 1 — Supplementary Information [file 41467_2018_6068_MOESM1_ESM.pdf]

SUPPLEMENTARY INFORMATION

Dynamics of cellular states of Fibro-Adipogenic Progenitors during myogenesis and muscle disease

Barbora Malecova, Sole Gatto, Usue Etxaniz, Magda Passafaro, Amy Cortez, Chiara Nicoletti, Lorenzo Giordani, Alessio Torcinaro, Marco De Bardi, Silvio Bicciato, Francesca De Santa, Luca Madaro, Pier Lorenzo Puri

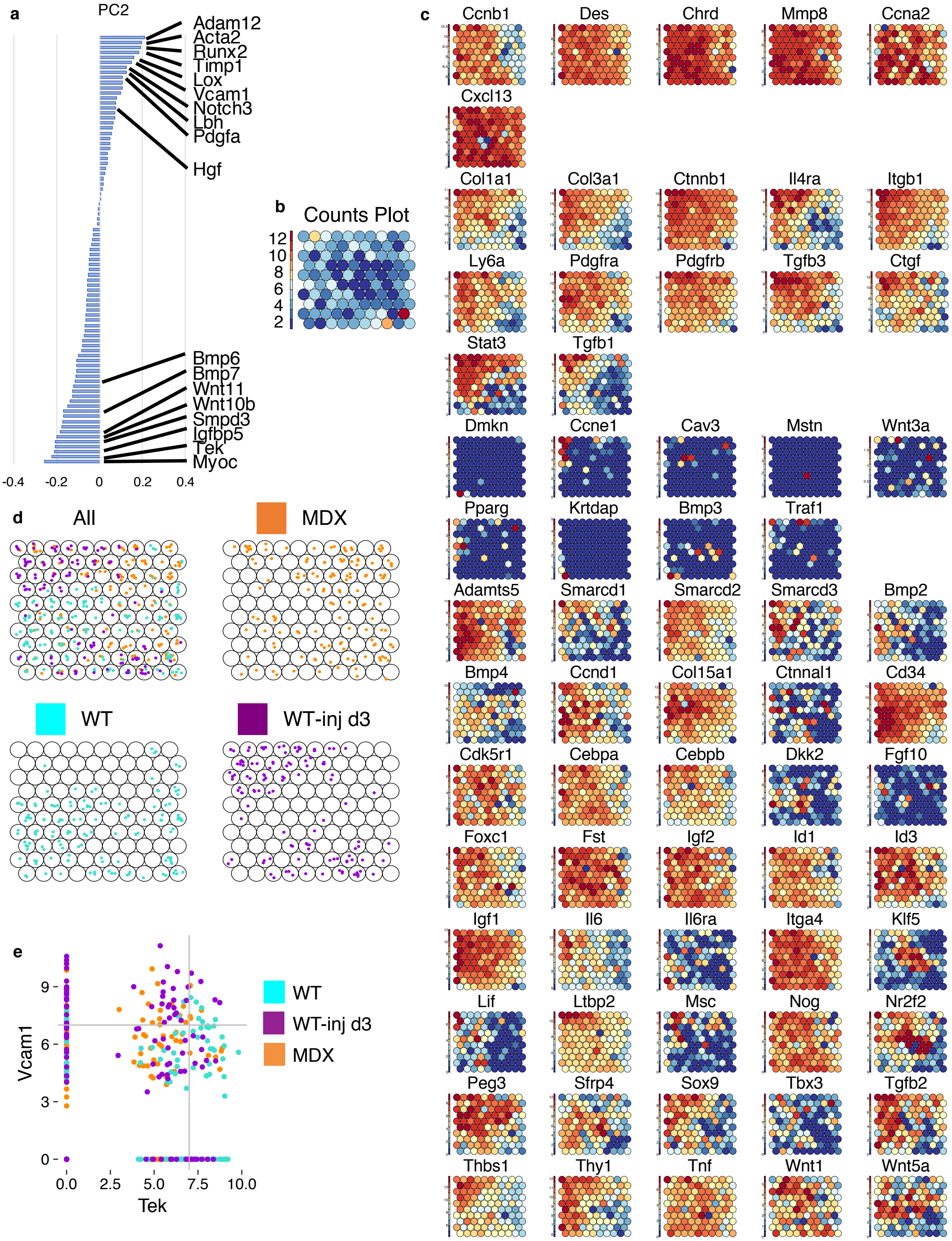
a
PC2
Adam12
Acta2
Runx2
Timp1
Lox
Vcam1
Notch3
Lbh
Pdgfa
Hgf
Bmp6
Bmp7
Wnt11
Wnt10b
Smpd3
Igfbp5
Tek
Myoc
-0.4
-0.2
0
0.2
0.4
b
Counts Plot
12
10
8
6
4
2
c
Ccnb1
Des
Chrd
Mmp8
Ccna2
Cxcl13
Col1a1
Col3a1
Ctnnb1
Il4ra
Itgb1
Ly6a
Pdgfra
Pdgfrb
Tgfb3
Ctgf
Stat3
Tgfb1
Dmkn
Ccne1
Cav3
Mstn
Wnt3a
Pparg
Krtdap
Bmp3
Traf1
Adamts5
Smarcd1
Smarcd2
Smarcd3
Bmp2
Bmp4
Ccnd1
Col15a1
Ctnnal1
Cd34
Cdk5r1
Cebpa
Cebpb
Dkk2
Fgf10
Foxc1
Fst
Igf2
Id1
Id3
Igf1
Il6
Il6ra
Itga4
Klf5
Lif
Ltbp2
Msc
Nog
Nr2f2
Peg3
Sfrp4
Sox9
Tbx3
Tgfb2
Thbs1
Thy1
Tnf
Wnt1
Wnt5a
d
All
MDX
WT
WT-inj d3
e
Vcam1
0
3
6
9
0.0
2.5
5.0
7.5
10.0
Tek
WT
WT-inj d3
MDX

Supplementary Figure 1 (related to figure 1). **Self Organizing Maps (SOM) clustering of FAPs** **a**. Principal component 2 sorted gene loadings. Genes showed in Figure 1B are highlighted here. **b**. Self Organizing Maps (SOM) scheme with colors representing the number of cells in each cluster (circle). **c**. SOM representation of gene expression in clusters of single FAP cells for all the genes in the analysis (except the ones shown in Fig. 1b). Expression is measured as Log_2Ex ($Log_2Ex=Ct_{(LOD)}-Ct_{(gene)}$) with LOD=24 (Limit Of Detection) and Ct=Cycle threshold. **d**. Distribution of each single cell color-coded for experimental condition in the SOM representation. **e**. Expression scatterplot of *Tek* and *Vcam1* gene expression. Cut-off is set at 7 Log_2Ex for both genes based on the SOM graph (Fig. 1b). The color coding represents the experimental conditions.

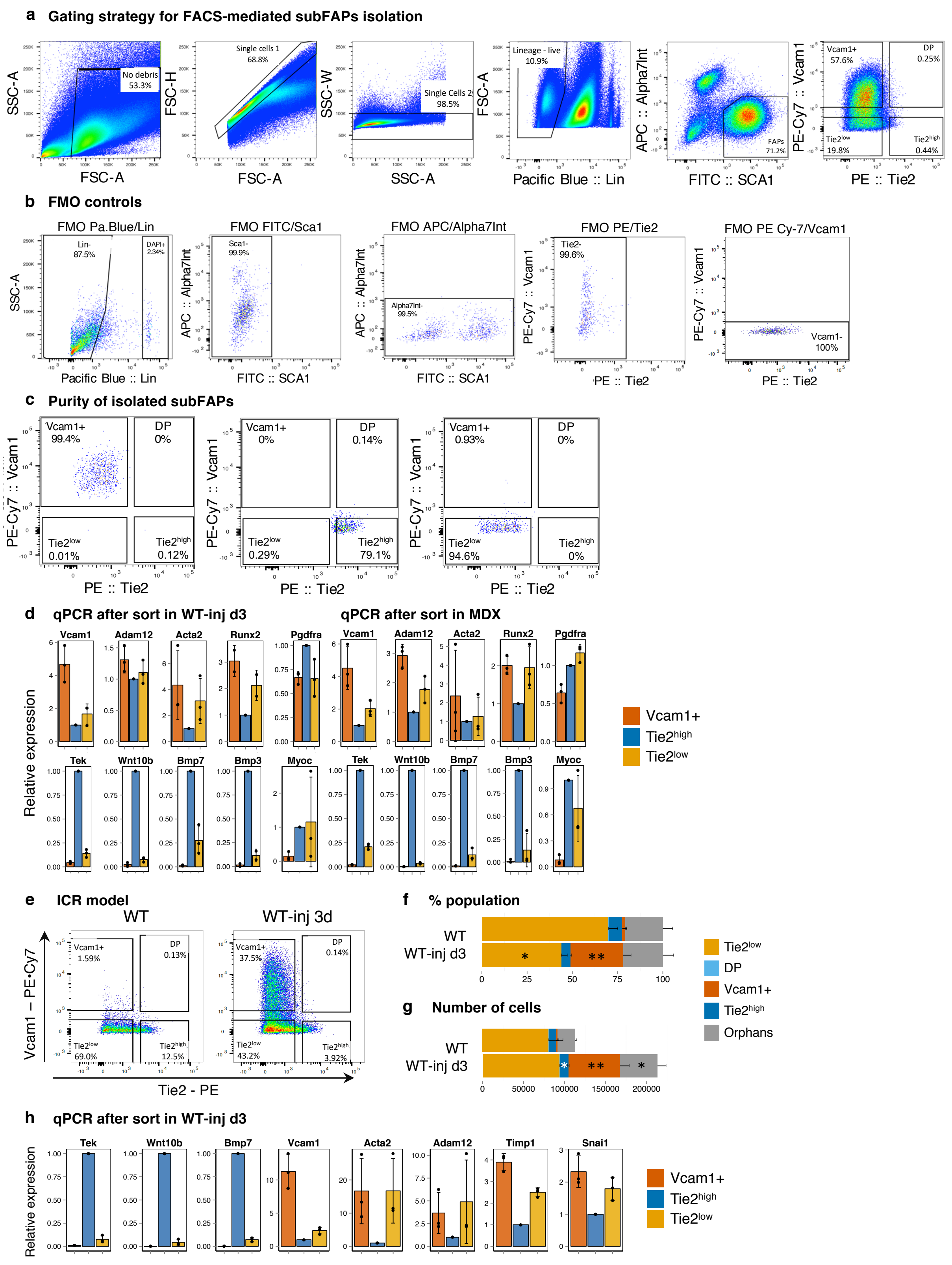
a Gating strategy for FACS-mediated subFAPs isolation
No debris 53.3%
Single cells 1 68.8%
Single Cells 2 98.5%
Lineage - live 10.9%
FAPs 71.2%
Vcam1+ 57.6%
DP 0.25%
Tie2low 19.8%
Tie2high 0.44%
SSC-A
FSC-A
FSC-H
SSC-W
Pacific Blue :: Lin
APC :: Alpha7Int
FITC :: SCA1
PE-Cy7 :: Vcam1
PE :: Tie2
b FMO controls
FMO Pa.Blue/Lin
Lin- 87.5%
DAPI+ 2.34%
FMO FITC/Sca1
Sca1- 99.9%
FMO APC/Alpha7Int
Alpha7Int- 99.5%
FMO PE/Tie2
Tie2- 99.6%
FMO PE Cy-7/Vcam1
Vcam1- 100%
c Purity of isolated subFAPs
Vcam1+ 99.4%
DP 0%
Tie2low 0.01%
Tie2high 0.12%
Vcam1+ 0%
DP 0.14%
Tie2low 0.29%
Tie2high 79.1%
Vcam1+ 0.93%
DP 0%
Tie2low 94.6%
Tie2high 0%
d qPCR after sort in WT-inj d3
qPCR after sort in MDX
Relative expression
Vcam1
Adam12
Acta2
Runx2
Pgdfra
Tek
Wnt10b
Bmp7
Bmp3
Myoc
Vcam1+
Tie2high
Tie2low
e ICR model
WT
WT-inj 3d
Vcam1+ 1.59%
DP 0.13%
Tie2low 69.0%
Tie2high 12.5%
Vcam1+ 37.5%
DP 0.14%
Tie2low 43.2%
Tie2high 3.92%
Vcam1 – PE•Cy7
Tie2 - PE
f % population
WT
WT-inj d3
Tie2low
DP
Vcam1+
Tie2high
Orphans
g Number of cells
WT
WT-inj d3
h qPCR after sort in WT-inj d3
Relative expression
Tek
Wnt10b
Bmp7
Vcam1
Acta2
Adam12
Timp1
Snai1
Vcam1+
Tie2high
Tie2low

Supplementary Figure 2 (related to figure 2). **FACS-based isolation and validation of subFAPs**

a. Representative FACS profiles showing the FACS strategy for Vcam1 and Tie2 based isolation of FAP subpopulations. **b**. FMO control for FACS-mediated FAPs isolation for setting of the gates. **c**. Purity of isolated FAPs subpopulations. **d.** Gene expression validation in FACS-isolated FAPs subpopulations by RT-PCR with primers listed in Supplementary Table 2 (mean + s.d., n=3; relative expression to Tie2 normalized to *Gapdh* gene). **e**. Representative FACS plots of FAPs expression of Tie2 and Vcam1 in wild type (WT) and WT notexin-injured day 3 (WT-inj d3) derived from *ICR*/HaJ mouse strain. FAPs were isolated from hind limb muscles. **f**. Distribution of subpopulations in each experimental condition after FACS sort (mean + s.e.m., n=3, Student's t-test, *p-value (P) < 0.05; **P < 0.01). **g**. Number of cells in each experimental condition belonging to the subpopulations after FACS sort (mean + s.e.m., n=3, Student's t-test, *P <0.05; **P <0.01). **h**. Gene expression validation in FACS-isolated FAPs subpopulations by RT-PCR with primers listed in Supplementary Table 2 (mean + s.d.; n=3; relative expression to Tie2 normalized to *Gapdh* gene).

See Supplementary Data 1 for the raw data and statistics for Supplementary Figures 2d, f, g and h.

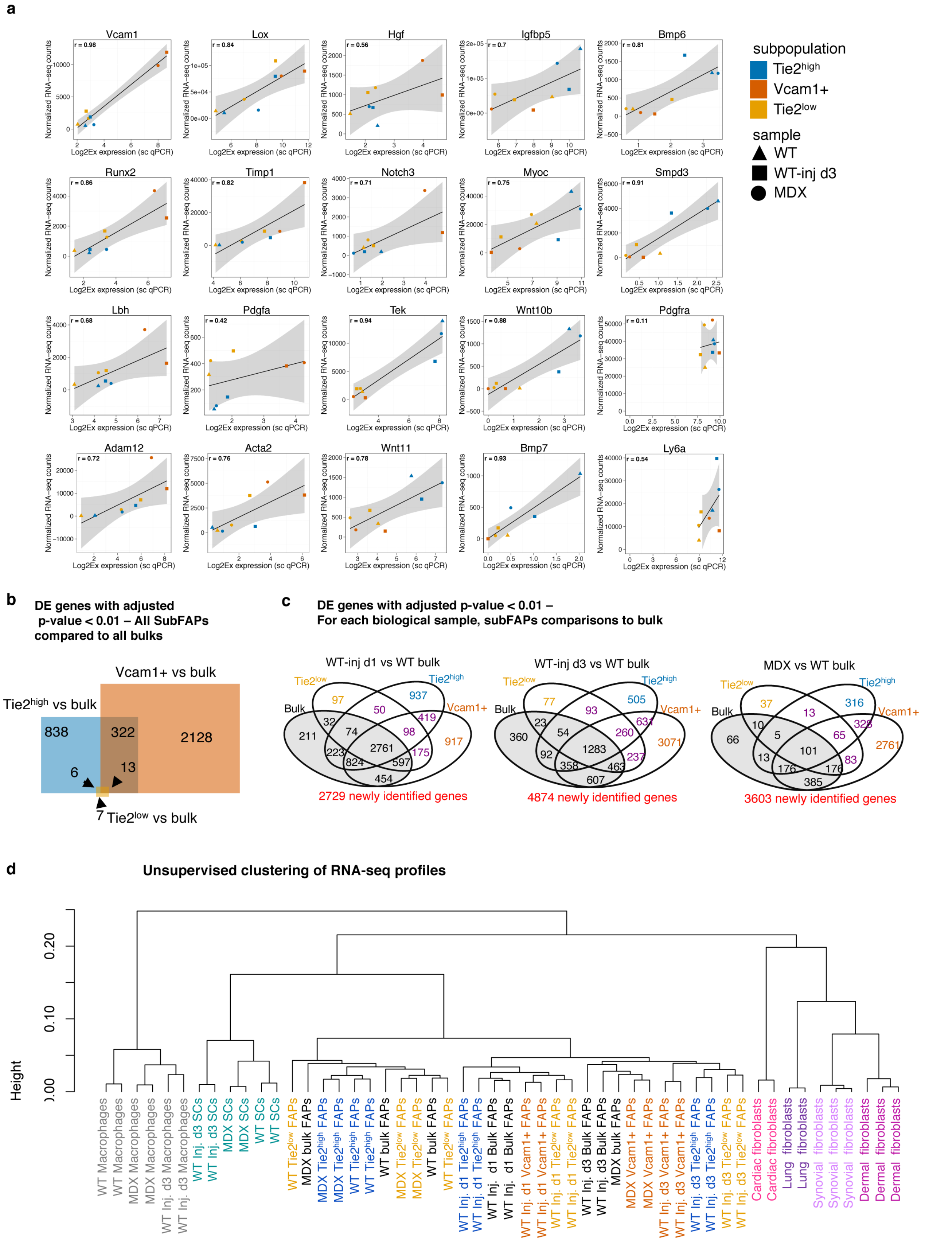
a
Vcam1
Lox
Hgf
Igfbp5
Bmp6
Runx2
Timp1
Notch3
Myoc
Smpd3
Lbh
Pdgfa
Tek
Wnt10b
Pdgfra
Adam12
Acta2
Wnt11
Bmp7
Ly6a
Normalized RNA−seq counts
Log2Ex expression (sc qPCR)
subpopulation
Tie2high
Vcam1+
Tie2low
sample
WT
WT-inj d3
MDX
b
DE genes with adjusted p-value < 0.01 – All SubFAPs compared to all bulks
Vcam1+ vs bulk
Tie2high vs bulk
Tie2low vs bulk
c
DE genes with adjusted p-value < 0.01 – For each biological sample, subFAPs comparisons to bulk
WT-inj d1 vs WT bulk
WT-inj d3 vs WT bulk
MDX vs WT bulk
2729 newly identified genes
4874 newly identified genes
3603 newly identified genes
d
Unsupervised clustering of RNA-seq profiles
Height

Supplementary Figure 3 (related to figure 3). **Transcriptome analysis of subFAPs**
a. Correlation between RNA-seq normalized counts and averaged single cell gene expression (Log2Ex) for each subpopulation in each experimental condition. **b.** Venn diagram showing the overlap among the differentially expressed genes in FAPs subpopulations compared to Bulk FAPs. **c.** Venn diagrams showing the overlap among the differentially expressed genes in FAP subpopulations in each treatment condition (WT-inj d1, WT-inj d3 and MDX) compared to bulk WT FAPs. **d.** Unsupervised clustering of various mouse cell types: bulk FAPs (black), subFAPs (Vcam1+ in brown, $Tie2^{high}$ in blue, $Tie2^{low}$ in gold), satellite cells (SC) (dark teal), macrophages (grey) in the different conditions (WT, MDX, injury day 1 and day 3) and mouse tissue-resident fibroblasts (cardiac in pink, lung in purple, synovial in lavender, dermal in plum).

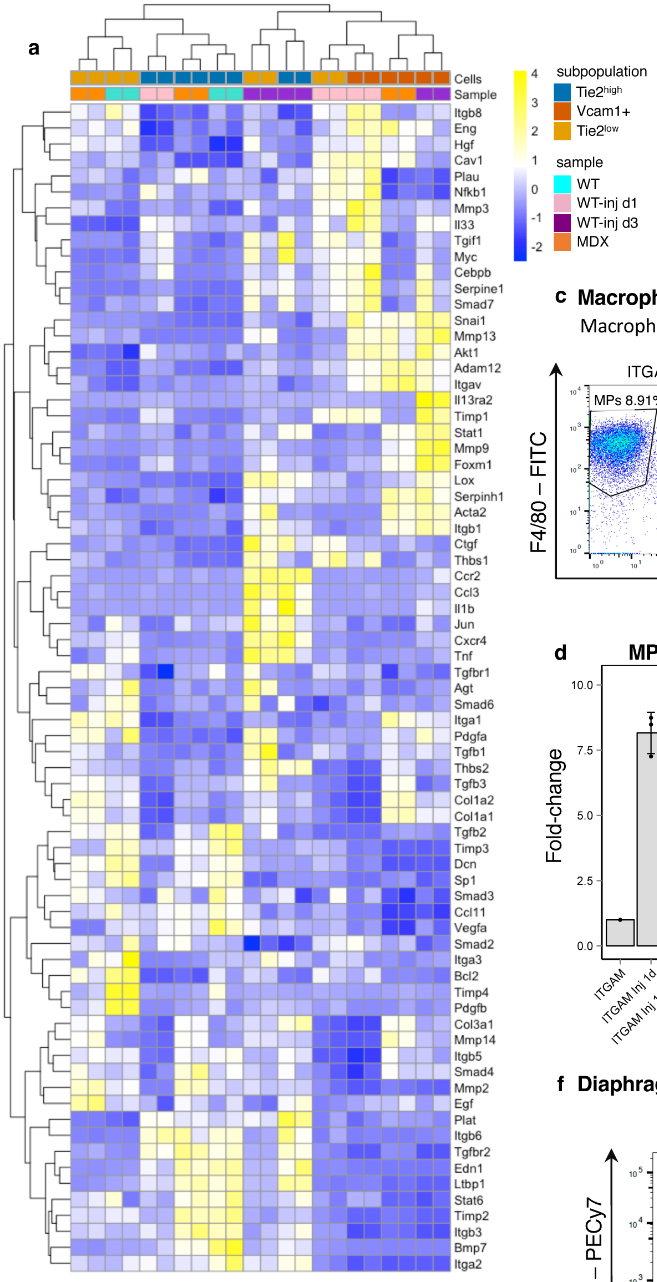
a
Cells
Sample
subpopulation
Tie2high
Vcam1+
Tie2low
sample
WT
WT-inj d1
WT-inj d3
MDX
Itgb8
Eng
Hgf
Cav1
Plau
Nfkb1
Mmp3
Il33
Tgif1
Myc
Cebpb
Serpine1
Smad7
Snai1
Mmp13
Akt1
Adam12
Itgav
Il13ra2
Timp1
Stat1
Mmp9
Foxm1
Lox
Serpinh1
Acta2
Itgb1
Ctgf
Thbs1
Ccr2
Ccl3
Il1b
Jun
Cxcr4
Tnf
Tgfbr1
Agt
Smad6
Itga1
Pdgfa
Tgfb1
Thbs2
Tgfb3
Col1a2
Col1a1
Tgfb2
Timp3
Dcn
Sp1
Smad3
Ccl11
Vegfa
Smad2
Itga3
Bcl2
Timp4
Pdgfb
Col3a1
Mmp14
Itgb5
Smad4
Mmp2
Egf
Plat
Itgb6
Tgfbr2
Edn1
Ltbp1
Stat6
Timp2
Itgb3
Bmp7
Itga2

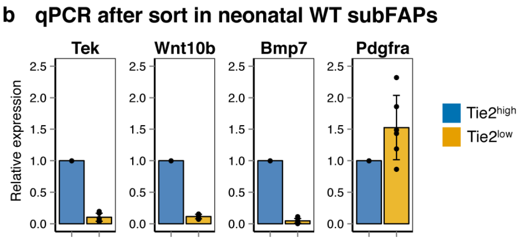
b qPCR after sort in neonatal WT subFAPs
Tek
Wnt10b
Bmp7
Pdgfra
Relative expression
Tie2high
Tie2low

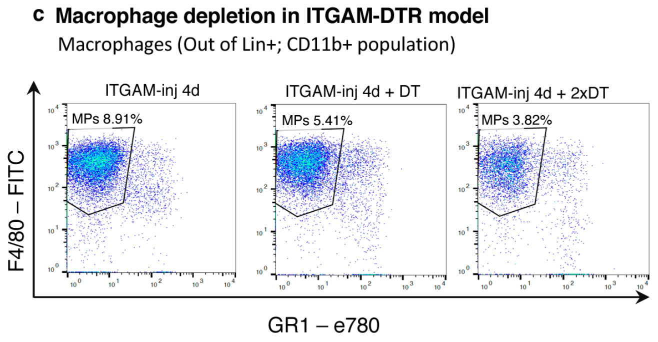
c Macrophage depletion in ITGAM-DTR model
Macrophages (Out of Lin+; CD11b+ population)
ITGAM-inj 4d
ITGAM-inj 4d + DT
ITGAM-inj 4d + 2xDT
MPs 8.91%
MPs 5.41%
MPs 3.82%
F4/80 – FITC
GR1 – e780

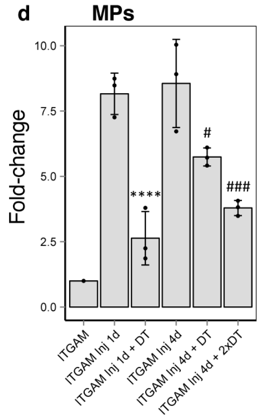
d MPs
Fold-change
****
#
###
ITGAM
ITGAM Inj 1d
ITGAM Inj 1d + DT
ITGAM Inj 4d
ITGAM Inj 4d + DT
ITGAM Inj 4d + 2xDT

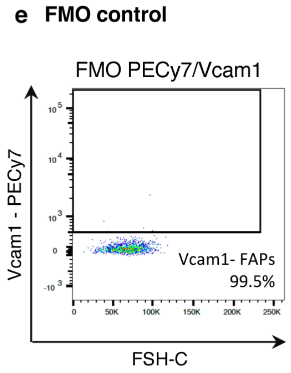
e FMO control
FMO PECy7/Vcam1
Vcam1 - PECy7
Vcam1- FAPs
99.5%
FSH-C

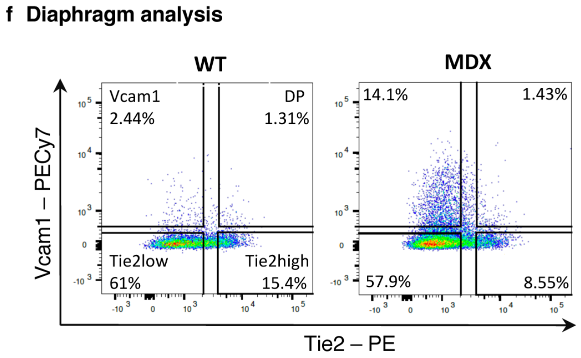
f Diaphragm analysis
WT
MDX
Vcam1
2.44%
DP
1.31%
Tie2low
61%
Tie2high
15.4%
14.1%
1.43%
57.9%
8.55%
Vcam1 – PECy7
Tie2 – PE

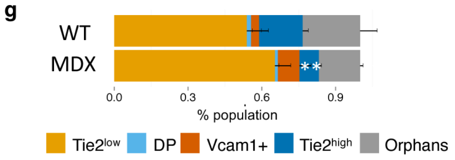
g
WT
MDX
**
% population
Tie2low
DP
Vcam1+
Tie2high
Orphans

Supplementary Figure 4 (related to figure 3 and 4). **Analysis of subFAPs in different experimental conditions**

a. Gene expression heatmap for fibrosis-related genes in the FAPs subpopulations. Gene expression is represented as z-score calculated across the rows. **b**. Gene expression validation in FACS-isolated neonatal FAPs subpopulations by RT-PCR with primers listed in Supplementary Table 2 (mean + s.d.; n=6; relative expression to Tie2 normalized to *Gapdh* gene). **c**. FACS-profile showing macrophage (MP) depletion 96h (4d) after acute muscle injury (inj) in single-injection and double-injection of diphtheria toxin (DT) versus PBS treated ITGAM mice. Macrophages were identified as F4/80+GR1- cells in the CD45+CD11b+ population. **d**. Quantification of macrophages by FACS 0, 24h (1d) and 96h (4d) days after an acute muscle injury in animals treated with single dose of DT, double-dose of DT or vehicle treated (Fold change based on the percentage of MPs out of live cells, ratio vs. control). Statistical significance was determined by one-way ANOVA with Bonferroni post hoc test, and only comparisons to the untreated groups ITGAM Inj.d1 (*) and ITGAM Inj.4d (#) are reported (mean + s.d., n=3 independent experiments, ANOVA, ****$P<0.0001$, #$P<0.05$, ###$P<0.001$). **e**. Vcam1 FMO control for the sort in Figure 4f. **f**. Representative FACS plots of FAPs expression of Tie2 and Vcam1 in wild type (WT) and mdx mice. FAPs were isolated from diaphragms. **g**. Distribution of subpopulations in each experimental condition by FACS (mean + s.d., n=3, Student's t-test, **$P < 0.01$).

See Supplementary Data 1 for the raw data and statistics for Supplementary Figures 4b, d and g.

Supplementary Table 1: List of Delta gene assays (Fluidigm) used in single cell gene expression profiling analysis.

Target Gene Symbol	Assay ID	Design RefSeq	Blast Hits	Gene Aliases	Gene Full Name	References
Acta2	GEA00043612	NM_007392.2	NM_007392.2	Actvs/a-SMA/SMalphaA/alphaSMA/0610041G09Rik	actin, alpha 2, smooth muscle, aorta	1
Adam12	GEA00049236	NM_007400.2	NM_007400.2	Mltna/mKIAA4001	a disintegrin and metallopeptidase domain 12 (meltrin alpha)	1,2
Adamts5	GEA00042705	NM_011782.2	NM_011782.2	ASMP-2/ADAMTS1/ADAM-TS5/ADAMTS11/AI481094/9530092O11Rik	a disintegrin-like and metallopeptidase (reprolysin type) with thrombospondin type 1 motif, 5 (aggrecanase-2)	3
Bmp2	GEA00012008	NM_007553.2	NM_007553.2	Bmp2a/AI467020	bone morphogenetic protein 2	4
Bmp3	GEA00012010	NM_173404.3	NM_173404.3	9130206H07/9530029I04Rik	bone morphogenetic protein 3	3
Bmp4	GEA00013254	NM_007554.2	NM_007554.2	Bmp-4/Bmp2b/Bmp2b1/Bmp2b-1	bone morphogenetic protein 4	
Bmp6	GEA00013255	NM_007556.2	NM_007556.2	Vgr1/D13Wsu115e	bone morphogenetic protein 6	3
Bmp7	GEA00013256	NM_007557.2	NM_007557.2	OP1	bone morphogenetic protein 7	1
Cav3	GEA00031914	NM_007617.2	NM_007617.2	Cav-3/M-cav/AI385751/M-caveolin/caveolin-3	caveolin 3	3
Ccna2	GEA00011724	NM_009828.2	NM_009828.2		cyclin A2	
Ccnb1	GEP00059972	NM_172301.3	NM_172301		cyclin B1	
Ccnd1	GEA00007896	NM_007631.2	NM_007631.2		cyclin D1	
Ccne1	GEA00011728	NM_007633.2	NM_007633.2		cyclin E1	

Target Gene Symbol	Assay ID	Design RefSeq	Blast Hits	Gene Aliases	Gene Full Name	References
Cd34	GEA00011730	NM_001111059.1	NM_133654.3/ NM_001111059.1	AU040960	CD34 antigen	5
Cdk5r1	GEA00012174	NM_009871.2	NM_009871.2	p25/p35/Cdk5r/ D11Bwg0379e	cyclin-dependent kinase 5, regulatory subunit 1 (p35)	3
Cebpa	GEA00011742	NM_007678.3	NM_007678.3	Cebp/CBF-A/C/ebpalpha	CCAAT/enhancer binding protein (C/EBP), alpha	
Cebpb	GEA00022507	NM_009883.3	NM_009883.3	LAP/LIP/CRP2/ NF-M/Nfil6/NF-IL6/IL-6DBP/C/EBPbeta	CCAAT/enhancer binding protein (C/EBP), beta	
Chrd	GEA00049273	NM_009893.2	NM_009893.2	Chd	chordin	
Col15a1	GEA00050330	NM_009928.3	NM_009928.3		collagen, type XV, alpha 1	3
Col1a1	GEA00011837	NM_007742.3	NM_007742.3	Cola1/Mov13/Cola-1/Mov-13/Col1a-1	collagen, type I, alpha 1	1
Col3a1	GEA00050479	NM_009930.2	NM_009930.2	Ms10w/Col3a-1/MMS10-W/AW550625/ mKIAA4231	collagen, type III, alpha 1	1
Ctgf	GEA00012830	NM_010217.2	NM_010217.2	Ccn2/Hcs24/Fisp12/fisp-12	connective tissue growth factor	1
Ctnnal1	GEA00048479	NM_018761.3	NM_018761.3	ACRP/C86009/ Catnal1/AI616177/AW545119	catenin (cadherin associated protein), alpha-like 1	3
Ctnnb1	GEA00011832	NM_007614.3	NM_001165902.1/NM_007614.3	Bfc/Mesc/Catnb	catenin (cadherin associated protein), beta 1	
Cxcl13	GEA00037774	NM_018866.2	NM_018866.2	BLC/Angie/BCA-1/BLR1L/ANGIE2/Scyb13/4631412M08Rik	chemokine (C-X-C motif) ligand 13	3
Des	GEA00011857	NM_010043.1	NM_010043.1		desmin	
Dkk2	GEA00022976	NM_020265.4	NM_020265.4		dickkopf homolog 2 (Xenopus laevis)	3

Target Gene Symbol	Assay ID	Design RefSeq	Blast Hits	Gene Aliases	Gene Full Name	References
Dmkn	GEA00048692	NM_001166173.1	NM_001166174.1/NM_001166173.1/NM_028618.2/NM_172899.4	SK30/SK89/cI-36/AW561900/C130074A08/1110014F24Rik	dermokine	3
Fgf10	GEA00013272	NM_008002.4	NM_008002.4	AEY17/Fgf-10/BB213776/Gsfaey17	fibroblast growth factor 10	3
Foxc1	GEA00033152	NM_008592.2	NM_008592.2	ch/Mf1/Mf4/Fkh1/fkh-1/FREAC3/frkhda	forkhead box C1	3
Fst	GEP00056734	NM_008046.2	NM_008046	AL033346	follistatin	3,6
Hgf	GEA00048519	NM_010427.4	NM_010427.4	NK1/NK2/HGF/SF/SF/HGF/C230052L06Rik	hepatocyte growth factor	
Id1	GEA00011760	NM_010495.2	NM_010495.2	Idb1/bHLHb24/AI323524/D2Wsu140e	inhibitor of DNA binding 1	
Id3	GEA00017854	NM_008321.2	NM_008321.2	Idb3/Hlh462/bHLHb25	inhibitor of DNA binding 3	
Igf1	GEA00012060	NM_001111274.1	NM_001111276.1/NM_001111275.1/NM_010512.4/NM_184052.3/NM_001111274.1		insulin-like growth factor 1	
Igf2	GEA00022766	NM_010514.3	NM_001122737.1/NM_001122736.1/NM_010514.3		insulin-like growth factor 2	
Igfbp5	GEA00038090	NM_010518.2	NM_010518.2	IGFBP-5/AI256729/AW208790/IGFBP-5P	insulin-like growth factor binding protein 5	3
Il4ra	GEA00045406	NM_001008700.3	NM_001008700.3		interleukin 4 receptor, alpha	
Il6	GEA00003392	NM_031168.1	NM_031168.1	Il-6	interleukin 6	3
Il6ra	GEA00011763	NM_010559.2	NM_010559.2	Il6r/CD126/IL-6R	interleukin 6 receptor, alpha	3
Itga4	GEA00022934	NM_010576.3	NM_010576.3	CD49D	integrin alpha 4	3
Itga7	GEA00049568	NM_008398.2	NM_008398.2	[a]7/alpha7	integrin alpha 7	5
Itgb1	GEA00012870	NM_010578.2	NM_010578.2	CD29/Fnrb/gpIIa/Gm9863/AA409975/AA960159/4633401G24Rik/ENSMUSG00000051907	integrin beta 1 (fibronectin receptor beta)	3

Target Gene Symbol	Assay ID	Design RefSeq	Blast Hits	Gene Aliases	Gene Full Name	References
Klf5	GEA00018009	NM_009769.4	NM_009769.4	CKLF/IKLF/Bteb2/4930520J07Rik	Kruppel-like factor 5	7
Krtdap	GEA00040820	NM_001033131.3	NM_001033131.3	Kdap/sk221	keratinocyte differentiation associated protein	3
Lbh	GEA00036846	NM_029999.4	NM_029999.4	1810009F10Rik/6720416L16Rik	limb-bud and heart	3
Lif	GEA00012874	NM_001039537.1	NM_008501.2/ NM_001039537.1		leukemia inhibitory factor	3
Lox	GEA00013188	NM_010728.2	NM_010728.2	rrg/TSC-160/AI893619	lysyl oxidase	
Ltbp2	GEA00013410	NM_013589.3	NM_013589.3	AW208642/MGC144212	latent transforming growth factor beta binding protein 2	3
Ly6a	GEA00022516	NM_010738.2	NM_010738.2	TAP/Sca1/Sca-1/Ly-6A.2/Ly-6A/E/Ly-6E.1	lymphocyte antigen 6 complex, locus A	5
Mmp8	GEA00044800	NM_008611.4	NM_008611.4	BB138268	matrix metallopeptidase 8	
Msc	GEA00035730	NM_010827.2	NM_010827.2	MyoR/bHLHa22	musculin	3
Mstn	GEA00022584	NM_010834.2	NM_010834.2	Cmpt/Gdf8	myostatin	
Myoc	GEA00036949	NM_010865.3	NM_010865.3	TIGR/GLC1A/AI957332	myocilin	3
Nog	GEA00011845	NM_008711.2	NM_008711.2		noggin	3
Notch3	GEA00012083	NM_008716.2	NM_008716.2	N3/hpbk/AW229011	notch 3	
Nr2f2	GEA00013339	NM_009697.3	NM_009697.3/ NM_183261.3	EAR3/ARP-1/SVP40/Aporp1/COUPTFB/COUP-TF2/Tcfcoup2/COUP-TFII/2700033K02Rik/9430015G03Rik	nuclear receptor subfamily 2, group F, member 2	3
Pdgfa	GEA00012905	NM_008808.3	NM_008808.3		platelet derived growth factor, alpha	3

Target Gene Symbol	Assay ID	Design RefSeq	Blast Hits	Gene Aliases	Gene Full Name	References
Pdgfra	GEA00009066	NM_001083316.1	NM_011058.2/NM_001083316.1	CD140a/Pdgfr-2/AI115593	platelet derived growth factor receptor, alpha polypeptide	3,8;
Pdgfrb	GEA00012906	NM_008809.2	NM_008809.2/NM_001146268.1	Pdgfr/CD140b/PDGFR-1/AI528809	platelet derived growth factor receptor, beta polypeptide	3
Pecam1	GEA00011820	NM_001032378.1	NM_001032378.1/NM_008816.2	Cd31/Pecam/C85791/PECAM-1	platelet/endothelial cell adhesion molecule 1	5
Peg3	GEA00044156	NM_008817.2	NM_008817.2	Pw1/End4/ASF-1/Gcap4/Zfp102/AL022617/mKIAA0287	paternally expressed 3	9,10
Pparg	GEA00011774	NM_011146.3	NM_001127330.1/NM_011146.3	Nr1c3/PPARgamma/PPAR-gamma/PPARgamma2/PPAR-gamma2	peroxisome proliferator activated receptor gamma	3
Runx2	GEA00011825	NM_001146038.1	NM_001145920.1/NM_009820.4/NM_001146038.1	Cbf/LS3/AML3/Osf2/Cbfa1/Cbfa-1/PEBP2aA/Pebp2a1/Pebpa2a	runt related transcription factor 2	7
Sfrp4	GEA00012110	NM_016687.3	NM_016687.3		secreted frizzled-related protein 4	3
Smarcd1	GEA00018090	NM_031842.2	NM_031842.2	Baf60a/D15Kz1/AA407987	SWI/SNF related, matrix associated, actin dependent regulator of chromatin, subfamily d, member 1	3
Smarcd2	GEA00018464	NM_031878.2	NM_001130187.1/NM_031878.2	Baf60b/AW322457	SWI/SNF related, matrix associated, actin dependent regulator of chromatin, subfamily d, member 2	3

Target Gene Symbol	Assay ID	Design RefSeq	Blast Hits	Gene Aliases	Gene Full Name	References
Smarcd3	GEA00046924	NM_025891.3	NM_025891.3	BAF60C/1500001J14Rik/2210409C08Rik	SWI/SNF related, matrix associated, actin dependent regulator of chromatin, subfamily d, member 3	3
Smpd3	GEA00044291	NM_021491.3	NM_021491.3	fro/nSMase2/AI427456/AW537966/4631433G07Rik	sphingomyelin phosphodiesterase 3, neutral	3
Sox9	GEA00037224	NM_011448.4	NM_011448.4	AV220920/mKIAA4243/2010306G03Rik	SRY-box containing gene 9	7
Stat3	GEA00003674	NM_213659.2	NM_213660.2/ NM_213659.2/ NM_011486.4	Aprf/AW109958/1110034C02Rik	signal transducer and activator of transcription 3	
Tbx3	GEA00018044	NM_198052.1	NM_198052.1/ NM_011535.2	D5Ertd189e	T-box 3	
Tek	GEA00012935	NM_013690.2	NM_013690.2	Hyk/Tie2/tie-2/Cd202b/AA517024	endothelial-specific receptor tyrosine kinase	4,8
Tgfb1	GEA00003840	NM_011577.1	NM_011577.1	Tgfb/Tgfb-1/TGFbeta1/TGF-beta1	transforming growth factor, beta 1	1
Tgfb2	GEA00022864	NM_009367.3	NM_009367.3	Tgfb-2/BB105277/Tgf-beta2	transforming growth factor, beta 2	1
Tgfb3	GEA00013326	NM_009368.3	NM_009368.3	Tgfb-3	transforming growth factor, beta 3	1
Thbs1	GEA00023044	NM_011580.3	NM_011580.3	TSP1/TSP-1/tbsp1/Thbs-1	thrombospondin 1	1
Thy1	GEA00013325	NM_009382.3	NM_009382.3		thymus cell antigen 1, theta	
Timp1	GEA00012943	NM_001044384.1	NM_011593.2/ NM_001044384.1	Clgi/Timp/TIMP-1	tissue inhibitor of metalloproteinase 1	3
Tnf	GEA00003864	NM_013693.2	NM_013693.2	DIF/Tnfa/TNFSF2/Tnfsf1a/TNFalpha/TNF-alpha	tumor necrosis factor	
Traf1	GEA00022533	NM_009421.3	NM_009421.3	4732496E14Rik	TNF receptor-associated factor 1	

Target Gene Symbol	Assay ID	Design RefSeq	Blast Hits	Gene Aliases	Gene Full Name	References
Vcam1	GEA00013392	NM_011693.3	NM_011693.3	CD106/Vcam-1	vascular cell adhesion molecule 1	
Wnt1	GEA00012118	NM_021279.4	NM_021279.4	sw/Int-1/Wnt-1/swaying	wingless-related MMTV integration site 1	5
Wnt10b	GEA00012122	NM_011718.2	NM_011718.2	Wnt12	wingless related MMTV integration site 10b	3
Wnt11	GEA00012124	NM_009519.2	NM_009519.2		wingless-related MMTV integration site 11	3
Wnt3a	GEA00012129	NM_009522.2	NM_009522.2	vt/Wnt-3a	wingless-related MMTV integration site 3A	5
Wnt5a	GEA00012131	NM_009524.2	NM_009524.2	Wnt-5a/8030457G12Rik	wingless-related MMTV integration site 5A	5

Supplementary Table 2: Sequences of the RT-PCR primers used in this study

**Mouse primers:**	**Sequences:**
Acta2-forward	GTCCCAGACATCAGGGAGTAA
Acta2-reverse	TCGGATACTTCAGCGTCAGGA
Adam12-forward	AGACGTGCTGACTGTGCAAC
Adam12-reverse	CCGTGTGATTTCGAGTGAGAGA
Bmp3-forward	ACTCCGTGAGACTGAGCCAA
Bmp3-reverse	CCTGTCATAGAGCCACAGCATA
Bmp7-forward	ACGGACAGGGCTTCTCCTAC
Bmp7-reverse	ATGGTGGTATCGAGGGTGGAA
Gapdh-forward	GCTCACTGGCATGGCCTTCCG
Gapdh-reverse	GTAGGCCATGAGGTCCACCAC
Myoc-forward	CTGCTTGGTGTGGGGAATG
Myoc-reverse	TCCCTTGGGCAGCTAGATTCA
Pdgfra-forward	GTCGTTGACCTGCAGTGGA
Pdgfra-reverse	CCAGCATGGTGATACCTTTGT
Runx2-forward	GACTGTGGTTACCGTCATGGC
Runx2-reverse	ACTTGGTTTTTCATAACAGCGGA
Snai1-forward	TGTGTGGAGTTCACCTTCCAG
Snai1-reverse	AGAGAGTCCCAGATGAGGGT
Tek-forward	GAGTCAGCTTGCTCCTTTATGG
Tek-reverse	AGACACAAGAGGTAGGGAATTGA
Timp1-forward	CGAGACCACCTTATACCAGCG
Timp1-reverse	ATGACTGGGGTGTAGGCGTA
Vcam1-forward	GCACTCTACTGCGCATCTT
Vcam1-reverse	CACCAGACTGTACGATCCTTTC
Wnt10b-forward	GAAGGGTAGTGGTGAGCAAGA
Wnt10b-reverse	GGTTACAGCCACCCCATTCC

Supplementary Table 3: RNA-seq datasets not generated in this study, used for unsupervised clustering analysis (related to Supplementary Fig. 3d).

Cell type	Strain	Age	Extraction Protocol	GEO ID	SRX ID	Ref.
Cardiac fibroblasts	FVB	8 weeks	Total RNA	GSM1223640	SRX344338	11
Cardiac fibroblasts	FVB	4 weeks	Total RNA	GSM1223641	SRX344339	11
Lung fibroblasts	C57BL/6J	Newborn	Poly(A)	GSM521651	SRX019276	12
Synovial fibroblasts	CBA x C57BL/6J	11 weeks	Total RNA	GSM2500874	SRX2583619	13
Synovial fibroblasts	CBA x C57BL/6J	11 weeks	Total RNA	GSM2500875	SRX2583620	13
Synovial fibroblasts	CBA x C57BL/6J	11 weeks	Total RNA	GSM2500876	SRX2583621	13
Dermal fibroblasts	FVB/N	6-8 weeks	Poly(A)	GSM2067698	SRX1594731	14
Dermal fibroblasts	FVB/N	6-8 weeks	Poly(A)	GSM2067699	SRX1594732	14
Dermal fibroblasts	FVB/N	6-8 weeks	Poly(A)	GSM2067700	SRX1594733	14
Satellite Cells	C57BL/6	8-12 weeks	Total RNA	-	SRR7075694	Iannoti et al., [in preparation]
Satellite Cells	C57BL/6	8-12 weeks	Total RNA	-	SRR7075695	Iannoti et al., [in preparation]
Satellite Cells	C57BL/6	8-12 weeks	Total RNA	-	SRR7075710	Iannoti et al., [in preparation]

Satellite Cells	C57BL/6	8-12 weeks	Total RNA	-	SRR7075711	Iannoti et al., [in preparation]
Satellite Cells	MDX	8-12 weeks	Total RNA	-	SRR7075698	Iannoti et al., [in preparation]
Satellite Cells	MDX	8-12 weeks	Total RNA	-	SRR7075699	Iannoti et al., [in preparation]
Macrophages	C57BL/6	8-12 weeks	Total RNA	-	SRR7075706	Iannoti et al., [in preparation]
Macrophages	C57BL/6	8-12 weeks	Total RNA	-	SRR7075707	Iannoti et al., [in preparation]
Macrophages	C57BL/6	8-12 weeks	Total RNA	-	SRR7075704	Iannoti et al., [in preparation]
Macrophages	C57BL/6	8-12 weeks	Total RNA	-	SRR7075705	Iannoti et al., [in preparation]
Macrophages	MDX	8-12 weeks	Total RNA	-	SRR7075708	Iannoti et al., [in preparation]
Macrophages	MDX	8-12 weeks	Total RNA	-	SRR7075709	Iannoti et al., [in preparation]

SUPPLEMENTARY REFERENCES

1. Uezumi, A. *et al.* Fibrosis and adipogenesis originate from a common mesenchymal progenitor in skeletal muscle. *Journal of Cell Science* **124,** 3654–3664 (2011).
2. Dulauroy, S. Di Carlo, S. E., Langa, F., Eberl, G. & Peduto, L. Lineage tracing and genetic ablation of ADAM12(+) perivascular cells identify a major source of profibrotic cells during acute tissue injury. *Nat. Med.* **18,** 1262–1270 (2012).
3. Saccone, V. *et al.* HDAC-regulated myomiRs control BAF60 variant exchange and direct the functional phenotype of fibro-adipogenic progenitors in dystrophic muscles. *Genes & Development* **28,** 841–857 (2014).
4. Wosczyna, M. N., Biswas, A. A., Cogswell, C. A. & Goldhamer, D. J. Multipotent progenitors resident in the skeletal muscle interstitium exhibit robust BMP-dependent osteogenic activity and mediate heterotopic ossification. *Journal of Bone and Mineral Research* **27,** 1004–1017 (2012).
5. Joe, A. W. B. *et al.* Muscle injury activates resident fibro/adipogenic progenitors that facilitate myogenesis. *Nature Cell Biology* **12,** 153–163 (2010).
6. Mozzetta C. *et al.* Fibroadipogenic progenitors mediate the ability of HDAC inhibitors to promote regeneration in dystrophic muscles of young, but not old Mdx mice. *EMBO Molecular Medicine* **5,** 626-639 (2013).
7. Smink J.J. & Leutz A. Instruction of mesenchymal stem cell fate by the transcription factor C/EBPβ. *Gene* **497,** 10-17 (2012)
8. Uezumi, A Fukada, S.-I., Yamamoto, N., Takeda, S. & Tsuchida, K. Mesenchymal progenitors distinct from satellite cells contribute to ectopic fat cell formation in skeletal muscle. *Nature Cell Biology* **12,** 143–152 (2010)
9. Mitchell, K. J. *et al.* Identification and characterization of a non-satellite cell muscle resident progenitor during postnatal development. *Nature Cell Biology* **12,** 257–266 (2010).
10. Pannérec, A., Formicola, L., Besson, V., Marazzi, G. & Sassoon, D. A. Defining skeletal muscle resident progenitors and their cell fate potentials. *Development* **140,** 2879–2891 (2013).
11. Giudice, J. *et al.* Alternative splicing regulates vesicular trafficking genes in cardiomyocytes during postnatal heart development. *Nature Communications* **5,** 3064 (2014)
12. Guttman, M. *et al.* Ab initio reconstruction of cell type-specific transcriptomes in mouse reveals the conserved multi-exonic structure of lincRNAs. *Nature Biotechnology* **28,** 503–510 (2010).
13. Ntougkos, E. *et al.* Genomic Responses of Mouse Synovial Fibroblasts During Tumor Necrosis Factor-Driven Arthritogenesis Greatly Mimic Those in Human Rheumatoid Arthritis. *Arthritis Rheumatol* **69,** 1588–1600 (2017).
14. Ruhland, M. K. *et al.* Stromal senescence establishes an immunosuppressive microenvironment that drives tumorigenesis. *Nature Communications* **7,** 11762 (2016).
