# Supplementary material for: Dynamics of cellular states of fibro-adipogenic progenitors during myogenesis and muscular dystrophy
Source: Nat Commun. 2018 Sep 10;9:3670. doi: 10.1038/s41467-018-06068-6 (PMC6131350; doi:10.1038/s41467-018-06068-6)
Supplement: Supplementary file 3 — Description of Additional Supplementary Files [file 41467_2018_6068_MOESM3_ESM.docx]

**Description of Additional Supplementary Files**

File Name: Supplementary Data 1

Description: Raw data and statistics related to supplementary figures.
